# Systematic review and mixed treatment comparison meta-analysis of randomized clinical trials of primary oral antifungal prophylaxis in allogeneic hematopoietic cell transplant recipients

**DOI:** 10.1186/s12879-015-0855-6

**Published:** 2015-03-17

**Authors:** Eric J Bow, David J Vanness, Monica Slavin, Catherine Cordonnier, Oliver A Cornely, David I Marks, Antonio Pagliuca, Carlos Solano, Lael Cragin, Alissa J Shaul, Sonja Sorensen, Richard Chambers, Michal Kantecki, David Weinstein, Haran Schlamm

**Affiliations:** CancerCare Manitoba, 675 McDermot Ave, Winnipeg, MB Canada; University of Wisconsin and Visiting Scientist at Evidera, Madison, Wisconsin USA; Royal Melbourne Hospital, Melbourne, Australia; Assistance Publique-Hopitaux de Paris, Hôpital Henri Mondor and Université Paris-Est-Créteil, Creteil, France; Department I of Internal Medicine, Clinical Trials Centre Cologne, ZKS Köln, BMBF 01KN1106, Center for Integrated Oncology CIO KölnBonn, Cologne Excellence Cluster on Cellular Stress Responses in Aging-Associated Diseases (CECAD), University of Cologne, Cologne, Germany; University Hospitals Bristol NHS Foundation Trust, Bristol, UK; King’s College Hospital, London, UK; Hospital Clínico, INCLIVA Foundation, University of Valencia, Valencia, Spain; Evidera, Bethesda, Maryland USA; Pfizer, Collegeville, Pennsylvania USA; Pfizer, Paris, France; HTS Pharma Consulting, New York, New York USA

**Keywords:** Antifungal, AlloHCT, Azoles, Invasive fungal infections, Mixed treatment comparison

## Abstract

**Background:**

Antifungal prophylaxis is a promising strategy for reducing invasive fungal infections (IFIs) in allogeneic hematopoietic cell transplant (alloHCT) recipients, but the optimum prophylactic agent is unknown. We used mixed treatment comparison (MTC) meta-analysis to compare clinical trials examining the use of oral antifungals for prophylaxis in alloHCT recipients, with the goal of informing medical decision-making.

**Methods:**

Randomized controlled trials (RCTs) of fluconazole, itraconazole, posaconazole, and voriconazole for primary antifungal prophylaxis were identified through a systematic literature review. Outcomes of interest (incidence of IFI/invasive aspergillosis/invasive candidiasis, all-cause mortality, and use of other antifungals) were extracted from eligible RCTs and incorporated into a Bayesian hierarchical random-effects MTC.

**Results:**

Five eligible RCTs, randomizing 2147 patients in total, were included. Relative to fluconazole, prophylaxis with itraconazole (odds ratio [OR]: 0.52; interquartile range [IQR]: 0.35–0.76), posaconazole (OR: 0.56; IQR: 0.32–0.99), and voriconazole (OR: 0.46; IQR: 0.28–0.73) reduced incidence of overall proven/probable IFI. Posaconazole (OR: 0.31; IQR: 0.17–0.58) and voriconazole (OR: 0.33; IQR: 0.17–0.58) prophylaxis reduced proven/probable invasive aspergillosis more than itraconazole (OR: 0.68; IQR: 0.42–1.12). All-cause mortality was similar across all mould-active agents.

**Conclusion:**

As expected, mould-active azoles prevented IFIs, particularly invasive aspergillosis, more effectively than fluconazole in alloHCT recipients. The paucity of comparative efficacy data suggests that other factors such as long-term tolerability, availability of intravenous formulations, local IFI epidemiology, and drug costs may need to form the basis for selection among the mould-active azoles.

**Electronic supplementary material:**

The online version of this article (doi:10.1186/s12879-015-0855-6) contains supplementary material, which is available to authorized users.

## Background

Invasive fungal infections (IFI) are a significant cause of morbidity and mortality in allogeneic hematopoietic cell transplant (alloHCT) recipients, with invasive mould infections due to *Aspergillus* spp. (invasive aspergillosis, IA) being most prevalent [1-4]. Early treatment strategies and antifungal prophylaxis are options for mitigating the impact of IFI in this population [5-7]. Although a meta-analysis published in 2007 concluded that antifungal prophylaxis reduced all-cause mortality, IFI-related mortality, and IFI incidence in alloHCT recipients [8], a more recent systematic review failed to demonstrate consistent treatment effects for these outcomes using direct and indirect comparisons [9]. For antifungal prophylaxis, particularly in the long-term outpatient setting, oral antifungals have the potential to be convenient and cost-effective [10,11]. However, the optimum oral agent for antifungal prophylaxis in alloHCT recipients post-transplant remains uncertain.

For physicians faced with the challenge of selecting a systemically active oral antifungal, the principal choices are fluconazole, which lacks anti-mould activity, and the mould-active agents itraconazole, posaconazole, and voriconazole. To our knowledge no single head-to-head randomized clinical trial (RCT) has directly compared more than 2 of these options in alloHCT recipients. The paucity of such studies impedes the use of traditional pairwise meta-analysis to inform the clinical decision-making process.

Network meta-analysis, can synthesize head-to-head comparisons of interventions not directly compared in clinical trials, as long as these interventions share one or more common comparators in a network of evidence. Furthermore, mixed treatment comparison (MTC) network meta-analyses allow for the combination of both direct and indirect evidence [12,13], and have been successfully employed to address similar questions in numerous clinical areas, including cardiovascular disease, osteoporosis, and bacterial infections [14-18], as well as for comparisons of different agents and strategies for antifungal treatment [19,20]. To extend a previously published traditional meta-analysis of antifungal prophylaxis [8], we conducted a systematic literature review and MTC of RCTs evaluating fluconazole, itraconazole, posaconazole, and voriconazole as primary antifungal prophylaxis in alloHCT recipients, including recently published trials. Our objective was to compare the efficacy of these agents for the prevention of documented IFI in alloHCT recipients based on several key outcomes, with the purpose of informing medical decision-making.

## Methods

### Systematic literature review

**Figure 1:**
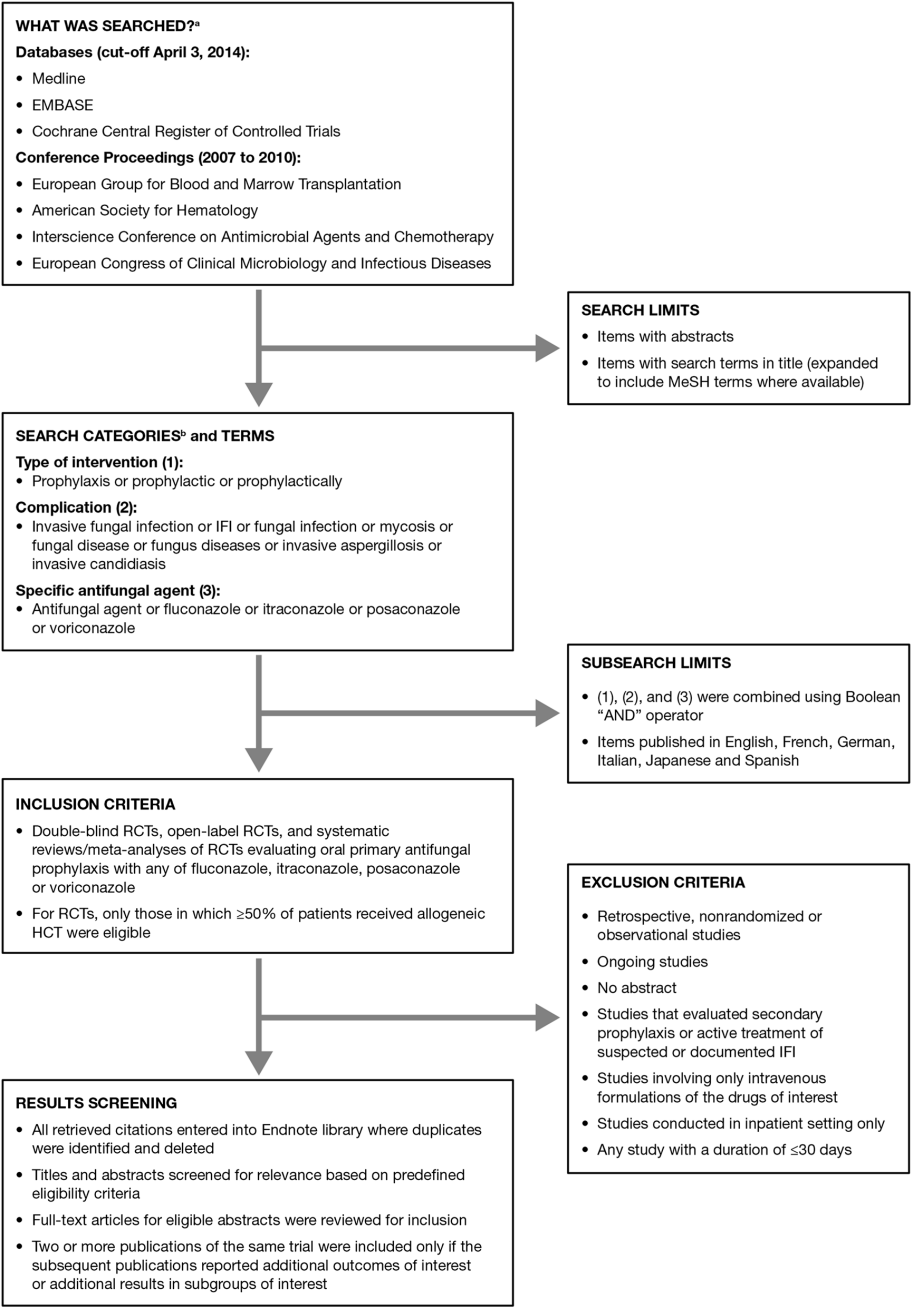
**Flow chart of the systematic literature review process and inclusion/exclusion criteria.**

We conducted a systematic literature review in 2014 to identify all relevant RCTs evaluating fluconazole, itraconazole, posaconazole, and voriconazole for primary oral antifungal prophylaxis in alloHCT recipients post-transplant. The search process and full inclusion/exclusion criteria are shown in Figure 1. The risk of bias across studies could not be assessed, since multiple studies assessing the same treatment effect were not available.

Only RCTs meeting the search criteria were included in the MTC network analysis of fluconazole, itraconazole, posaconazole, and voriconazole if they included a comparator common to multiple RCTs. For example, the hypothetical common comparator “C” can indirectly link comparators of interest “A” and “B” in Trial 1 comparing interventions “A” versus “C” and Trial 2 comparing “B” versus “C”. However, Trial 3 comparing “B” versus “D” would not be included in the hypothetical “A” versus “B” network since comparator “D” is neither a comparator of interest nor a common comparator that can indirectly inform an “A” versus “B” comparison.

### Outcomes evaluated

The main outcome of interest was the incidence of proven/probable IFI using the consensus criteria described by Ascioglu *et al.* [21]. Other outcomes were all-cause mortality, proven/probable IA, proven invasive candidiasis (IC), and administration of other licensed antifungal therapy (OLAT; defined as any systemic antifungal other than randomized study drug, including a temporary switch to an intravenous agent in patients not tolerating oral prophylaxis). The cumulative proportions of patients for each of these outcomes at 180 days post-transplant (or the closest available time point) were extracted from each RCT. Data extraction was performed in duplicate by 2 of the authors (LC, AJS) and the extracted data were independently reviewed by 2 additional authors (EJB, SS).

### Quantitative data synthesis

We used Bayesian hierarchical random-effects MTCs, estimated with Markov Chain Monte Carlo methods (WinBUGS v.1.4.3), to estimate posterior distributions for the comparative effectiveness of the interventions for each of the extracted outcomes [22]. These posterior distributions estimate the probability, given available evidence and a specified statistical model, that the comparative effectiveness takes on a particular value or lies within a specified range of values. For example, the probability is 75% (3:1 odds) that the true value of an odds ratio lies below the upper bound of the posterior interquartile range. A central 95% credible interval is comparable to a classical 95% confidence interval but, unlike the classical confidence interval, can be interpreted as having 95% probability of containing the true value. The probability is 97.5% (39:1 odds) that the true value of an odds ratio lies below the upper bound of the central 95% credible interval.

Posterior probabilities can also be used to estimate the probability that one treatment is more effective than another for each outcome, an advantage for informing decision-makers in cases where traditional thresholds of statistical significance (ie, 95%) cannot be achieved using standard methods [14]. In this MTC, we estimated the probability that each treatment resulted in a lower incidence than fluconazole for the respective outcome and the probability for each treatment to have the lowest incidence of all treatments (including fluconazole) for the respective outcome to help identify the most effective antifungal.

### Model selection

We used a conservative unconstrained baseline approach to account for potential between-trial heterogeneity in the risk of IFI on baseline treatment (ie, fluconazole) [14,18,23]. Our random effect specification for the treatment effect parameters assumed that the included trials are similar enough, both clinically and methodologically, that the estimated differences in treatment efficacy (in this case all-cause mortality, IFI risk, and OLAT use for each comparator relative to fluconazole) are “exchangeable” or similar across trials [13]. While we expected baseline infection risks to differ among trials according to timeframe and clinical context, we had no a priori expectations for systematic differences in relative risks by treatment.

Bayesian meta-analysis allows for uncertainty in the amount of heterogeneity of treatment effects between studies. We followed standard approaches for assigning a non-informative prior to the heterogeneity parameter. However, in cases such as our analysis, where MTCs include a relatively small number of studies, results can be sensitive to the choice of prior distribution for the parameter estimating the degree of heterogeneity [24]. Furthermore, the use of a non-informative prior when the number of included studies is small can fail to rule out unrealistically large degrees of heterogeneity. Therefore, we conducted a post-hoc sensitivity analysis based on previously published empirical Bayesian methods, which used variation in observed pairwise treatment effectiveness estimates to provide a modestly informative prior for heterogeneity [24,25]. Full details of the statistical analysis and model code are provided in the online Additional file 1.

## Results

### Summary of included studies

**Figure 2:**
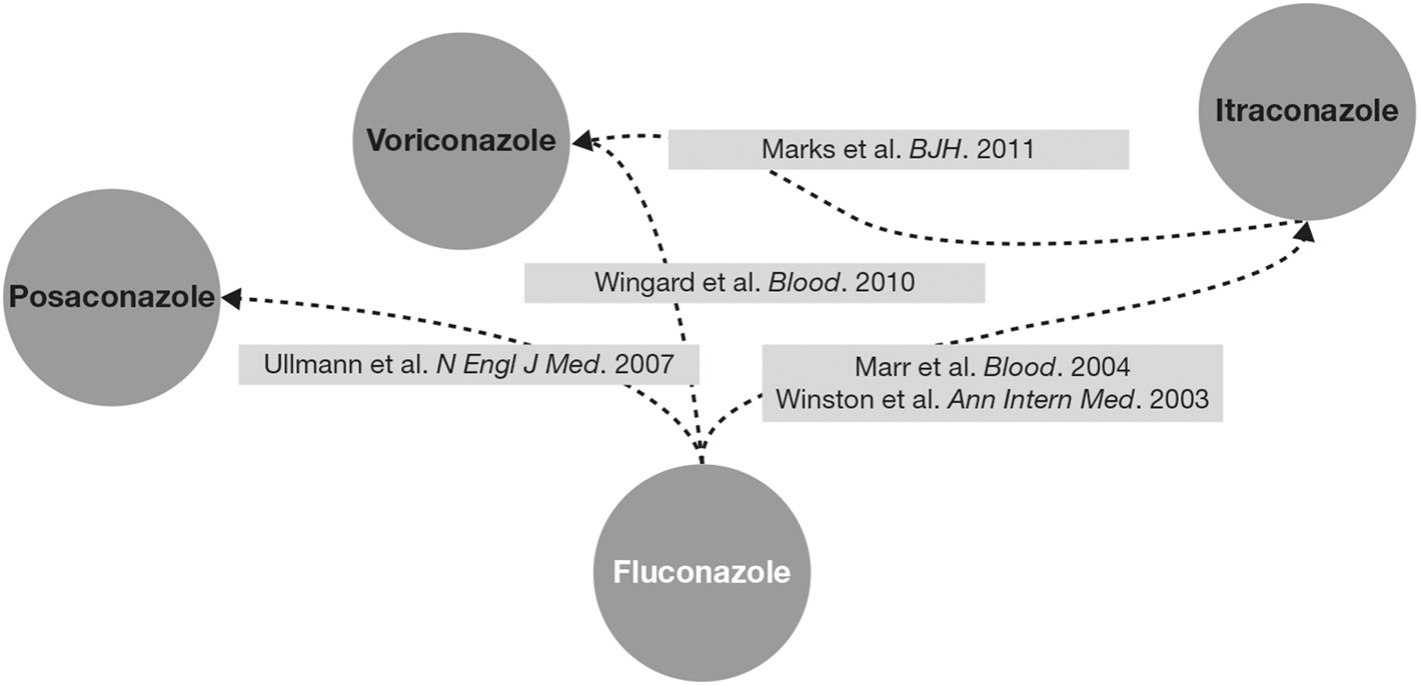
**Evidence network of randomized controlled trials (RCTs) included into the mixed treatment comparison (MTC).**

The systematic literature search identified 5 published RCTs that met predefined criteria for inclusion into the MTC [10,26-29]. Our literature search identified 6 additional RCTs that were not able to directly or indirectly inform a comparison of fluconazole, itraconazole, posaconazole, and voriconazole [30-35]. A flow chart of search results and a summary of each trial’s characteristics and patient populations are shown in the online Additional file 1. The 5 head-to-head studies [10,26-29] constituting the evidence network (Figure 2) for the MTC randomized a total of 2147 patients, with individual study sample sizes ranging from 140–600 patients. Four studies were multicentre trials, 3 studies had an open-label design, and 2 had a double-blind design. Fluconazole (with a total of 813 randomized patients) was a comparator in 4 RCTs, itraconazole (n = 485) was a comparator in 3, voriconazole (n = 548) in 2, and posaconazole (n = 301) was a comparator in a single trial. The data extracted from these studies for each outcome are shown in Table 1. The overall estimates of heterogeneity were large, particularly in the main analysis using a non-informative prior (see online Additional file 1).

**Table 1:** **Outcomes extracted from the randomized clinical trials and included into the mixed treatment comparison**

**Study**	**All-cause mortality**	**Incidence of proven/probable IFI overall**	**Incidence of proven/probable IA**	**Incidence of proven IC**	**Incidence of OLAT use**
**Winston 2003** [29]					
Fluconazole	28/67 (42%)	17/67 (25%)	8/67 (12%)	8/67 (12%)	Not reported
Itraconazole	32/71 (45%)	6/71 (8%)	3/71 (4%)	2/71 (3%)	Not reported
**Marr 2004** [26]					
Fluconazole	44/148 (30%)	25/148 (17%)	20/148 (14%)	5/148 (3%)	25/148 (17%)
Itraconazole	55/151 (36%)	19/151 (13%)^a^	16/151 (11%)	4/151 (3%)	19/151 (13%)
**Ullmann 2007** [27]					
Fluconazole	59/299 (20%)	27/299 (9%)	21/299 (7%)	4/299 (1%)	29/288 (10%)
Posaconazole	58/301 (19%)	16/301 (5%)	7/301 (2%)	4/301 (1%)	31/291 (11%)
**Wingard 2010** [28]					
Fluconazole	59/295 (20%)	24/295 (8%)	17/295 (6%)	5/295 (2%)	89/295 (30%)
Voriconazole	57/305 (19%)	14/305 (5%)	9/305 (3%)	3/305 (1%)	73/305 (24%)
**Marks 2011** [10]					
Itraconazole	44/241 (18%)	5/241 (2%)	5/241(2%)	0/241 (0%)	101/241 (42%)
Voriconazole	40/224 (18%)	3/224 (1%)	1/224 (0.4%)	2/224 (1%)	67/224 (30%)

IFI, invasive fungal infections; IA, invasive aspergillosis; IC, invasive candidiasis; OLAT, other licensed antifungal therapy.

^a^One patient developed both proven IC and probable IA, which was counted as a single IFI instead of 2 separate IFIs.

### MTC results

Based on the MTC estimates, voriconazole was the agent most likely to reduce incidence of overall proven/probable IFI at 180 days post-transplant relative to fluconazole, closely followed by itraconazole and posaconazole; this conclusion was reflected in the posterior probability of having a lower IFI risk than fluconazole and the posterior probability of having the lowest IFI risk among the 4 agents, both of which were most favourable with voriconazole (Table 2). However, in the base-case analysis, none of the risk differences in this outcome, nor any of the other outcomes (below), reached statistical significance at the standard 95% level – ie, all posterior probabilities of differences in outcomes were found to be less than 0.95.

**Table 2:** **Estimated treatment effect for each outcome relative to fluconazole (base case analysis using a non-informed prior)**

**Comparator**	**Median posterior odds-ratio relative to fluconazole ** **(interquartile range)** ^**a**^	**Posterior probability of having lower incidence than fluconazole**	**Posterior probability of having the lowest incidence of all treatments**
**Proven/probable IFI at 180 days**			
Fluconazole	–	–	2%
Itraconazole	0.52 (0.35–0.76)	84%	27%
Posaconazole	0.56 (0.32–0.99)	75%	32%
Voriconazole	0.46 (0.28–0.73)	84%	39%
**Proven/probable IA at 180 days**			
Fluconazole	–	–	2%
Itraconazole	0.68 (0.42–1.12)	71%	9%
Posaconazole	0.31 (0.15–0.63)	83%	47%
Voriconazole	0.33 (0.17–0.58)	87%	41%
**Proven IC at 180 days**			
Fluconazole	–	–	5%
Itraconazole	0.28 (0.11– 0.60)	85%	59%
Posaconazole	0.98 (0.28–3.45)	51%	23%
Voriconazole	1.19 (0.43–4.19)	46%	13%
**All-cause mortality**			
Fluconazole	–	–	20%
Itraconazole	1.18 (0.96–1.44)	29%	11%
Posaconazole	0.98 (0.74–1.27)	53%	40%
Voriconazole	1.02 (0.82–1.26)	48%	29%
**OLAT use at 180 days**			
Fluconazole	–	–	10%
Itraconazole	0.91 (0.49–1.58)	56%	18%
Posaconazole	1.08 (0.53–2.21)	46%	23%
Voriconazole	0.63 (0.35–1.09)	73%	49%

IFI, invasive fungal infections; IA, invasive aspergillosis; IC, invasive candidiasis; OLAT, other licensed antifungal therapy.

^a^Estimates less than zero indicate a reduced probability of proven/probable IFI at 180 days relative to fluconazole.

MTC estimates suggested that voriconazole and posaconazole were associated with the greatest reduction in probability of proven/probable IA by day 180 post-transplant relative to fluconazole, followed by itraconazole (Table 2). Voriconazole prophylaxis was more likely to yield a lower risk of proven/probable IA by day 180 relative to fluconazole (probability of 87%) than either posaconazole (83%) or itraconazole (71%). Posaconazole had a higher posterior probability of having the lowest IA risk by day 180 post-transplant (47%) than voriconazole (41%); the respective probability was only 9% for itraconazole and 2% for fluconazole.

Itraconazole had the most favourable estimated treatment effect on the prevention of proven IC at 180 days post-transplant relative to fluconazole, while posaconazole and voriconazole appeared to have a similar treatment effect to fluconazole (Table 2). In terms of avoiding the need to use OLAT, voriconazole had the most favourable estimated treatment effect, based on the observation that its posterior probability of having the lowest incidence of OLAT use (ie, 49%) was about 2–5 times higher than that for the other 3 agents; for this outcome, the probability that voriconazole was better than fluconazole was 73%, ie, about 20% higher than for itraconazole and 30% higher than for posaconazole (Table 2). There were no noteworthy differences between any of the azoles in all-cause mortality (Table 2).

**Figure 3:**
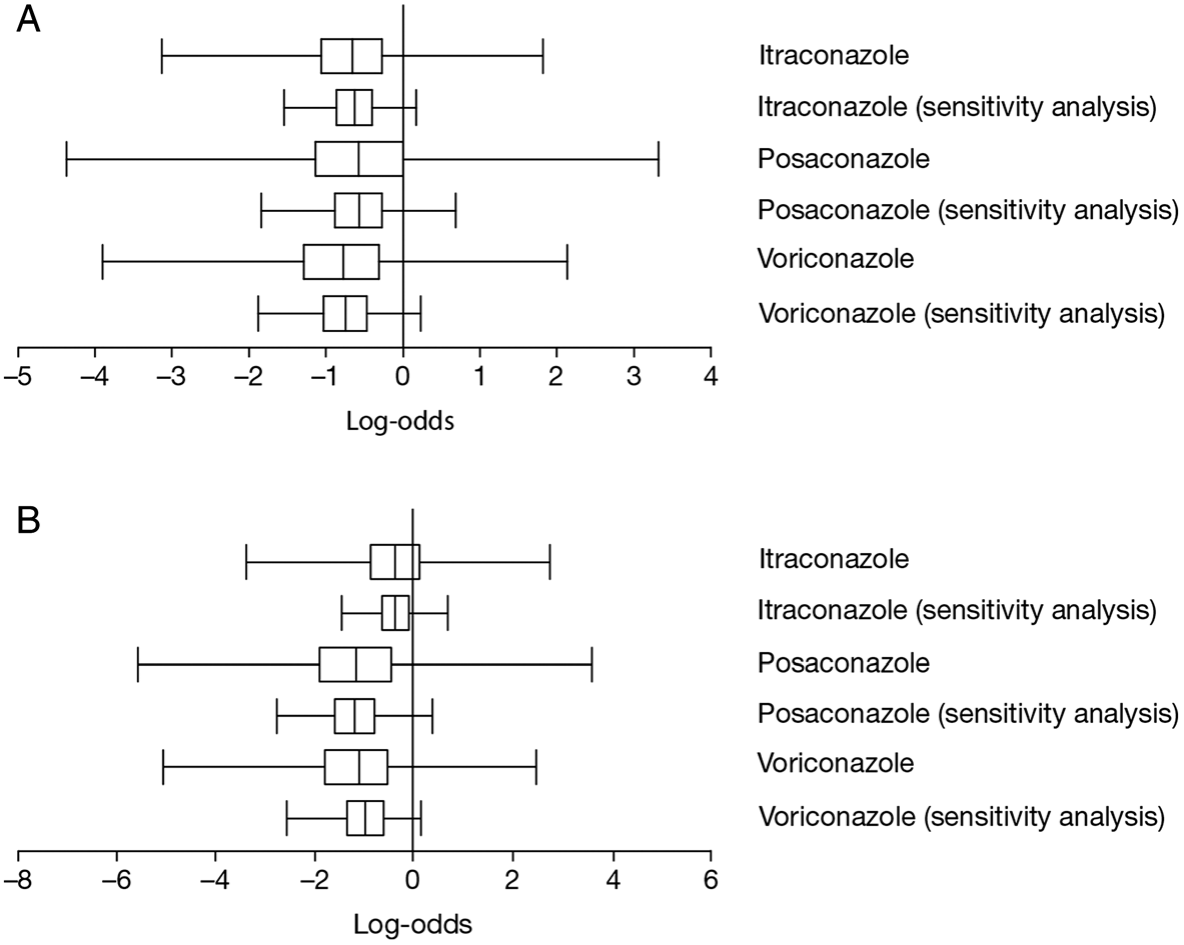
**Treatment effect of antifungal agents on A) proven/probable invasive fungal infection (IFI) and B) proven/probable invasive aspergillosis (IA) at 180 days, compared between the base-case mixed treatment comparison (MTC) and the sensitivity analysis MTC using an empirical prior, expressed in log odds.** Estimates less than zero indicate a reduced probability of IFI relative to fluconazole. The vertical bar of the box plot represents the posterior median value (probability <50%); the outer limits of the box plot represent the posterior interquartile range (probability 25%–75%); whiskers represent the most extreme Markov Chain Monte Carlo values of the posterior no more than 1.5 times the width of the interquartile range above or below the upper or lower bounds of the interquartile range.

The post-hoc sensitivity analysis using an empirical Bayesian prior for the heterogeneity parameter (see Methods section for details), yielded estimated treatment effects comparable to the base case (Table 3). However, credible intervals in this additional analysis were considerably less wide than in the base case; to illustrate this point, estimated posterior credible intervals (on the log-odds scale) for IFI overall and IA from both analyses are depicted in Figure 3A and B, respectively. Some of the notable differences in posterior probabilities observed in the base-case analysis became even more pronounced in the sensitivity analysis: for instance, the probability of itraconazole and voriconazole being better than fluconazole for prevention of IFI and the probability of posaconazole and voriconazole being better than fluconazole for prevention of IA were now found to be ≥95% and thus reached traditional thresholds of statistical significance. Results for all outcomes were very similar in a sensitivity analysis that excluded the single posaconazole RCT (see online Additional file 1), and was therefore limited to fluconazole, itraconazole, and voriconazole.

**Table 3:** **Estimated treatment effect for each outcome relative to fluconazole (sensitivity analysis using an empirical prior)**

**Comparator**	**Median posterior odds-ratio relative to fluconazole ** **(interquartile range)** ^**a**^	**Posterior probability of having lower incidence than fluconazole**	**Posterior probability of having the lowest incidence of all treatments**
**Proven/probable IFI at 180 days**			
Fluconazole	–	–	0%
Itraconazole	0.54 (0.42–0.67)	95%	27%
Posaconazole	0.57 (0.41–0.76)	88%	29%
Voriconazole	0.47 (0.36–0.63)	95%	44%
**Proven/probable IA at 180 days**			
Fluconazole	–	–	0%
Itraconazole	0.68 (0.53–0.90)	82%	4%
Posaconazole	0.30 (0.20–0.45)	96%	59%
Voriconazole	0.37 (0.26–0.54)	96%	36%
**Proven IC at 180 days**			
Fluconazole	–	–	2%
Itraconazole	0.31 (0.19–0.49)	95%	71%
Posaconazole	0.97 (0.49–1.96)	51%	16%
Voriconazole	0.90 (0.49–1.69)	55%	12%
**All-cause mortality**			
Fluconazole	–	–	22%
Itraconazole	1.18 (1.03–1.34)	21%	7%
Posaconazole	0.98 (0.82–1.16)	54%	41%
Voriconazole	1.01 (0.88–1.17)	48%	30%
**OLAT use at 180 days**			
Fluconazole	–	–	3%
Itraconazole	0.95 (0.78–1.14)	58%	7%
Posaconazole	1.07 (0.83–1.37)	43%	12%
Voriconazole	0.64 (0.54–0.75)	94%	79%

IFI, invasive fungal infections; IA, invasive aspergillosis; IC, invasive candidiasis; OLAT, other licensed antifungal therapy.

^a^Estimates less than zero indicate a reduced probability of proven/probable IFI at 180 days relative to fluconazole.

## Discussion

Transplant physicians are frequently faced with the difficult choice of selecting the most appropriate and efficacious option for oral antifungal prophylaxis in alloHCT recipients. In the absence of a large, multi-arm RCT comparing all systemically active oral antifungals, network meta-analyses can provide relevant information to help guide health intervention decision-making; this methodology is increasingly utilized for similar purposes across therapeutic areas [12,36].

Bayesian statistical inference differs from classical statistics in that it provides probability distributions for treatment effects, expressed as posterior credible intervals (rather than confidence intervals) [37]. One advantage of using Bayesian credible intervals is that they can be more intuitively interpreted in terms of the probability that relative efficacy lies within a specific range. Bayesian methods, therefore, allow us to directly report the probabilities of outperforming fluconazole or of being the best agent overall for each mould-active azole, which physicians can then factor into the selection process [38].

While our base-case MTC analysis allowed for substantial heterogeneity across the studies, our conservative approach came at the cost of a reduced statistical inference and wider credible intervals, thereby reducing our ability to detect actual differences between treatments. Thus, the available data did not allow our base-case MTC to distinguish between itraconazole, posaconazole, and voriconazole when using the 5% threshold for type-I error typically employed in classical hypothesis testing. Our sensitivity analysis using an empirical prior yielded some comparisons that did meet the traditional 5% threshold, ie, itraconazole and voriconazole had lower IFI risk and posaconazole and voriconazole had lower IA risk than fluconazole. Regardless, our objective was not to test causal research hypotheses (when prespecified confidence thresholds are useful), but rather to help decision-makers compare the efficacy of different interventions [39,40].

Although regulatory authorities will always prefer a high degree of certainty for these comparisons, the requirement for a specific threshold of “confidence” (typically 95%) may result in sub-optimal outcomes in situations where a treatment decision cannot be deferred [41,42]. Such is the case for antifungal prophylaxis in alloHCT recipients: while the most efficacious oral agent is currently unknown, a choice must still be made in those patients who are deemed to benefit from a prophylactic approach. The probabilities of superiority estimated by MTC represent an objective measure of comparative efficacy that can be taken into account when making this choice.

We note that our post-hoc empirical Bayesian sensitivity analysis did achieve statistical significance at the standard 95% level in several of the important outcomes: the posterior probabilities of itraconazole/voriconazole being superior to fluconazole for prevention of IFI overall, posaconazole/voriconazole being better than fluconazole for prevention of IA, and itraconazole being better than fluconazole for prevention of IC. The probability of voriconazole being superior to fluconazole for the reduction of OLAT use was found to be 94% in this sensitivity analysis. Empirical Bayesian methods are sometimes criticized for “using the data twice” [43], which is the reason this approach was not chosen for the base-case analysis. However, this method can improve statistical inference and has previously been shown to provide accurate inference in random effects meta-analysis [24].

Based on estimated probabilities of superiority, our analyses suggest that broad-spectrum mould-active azoles are more effective than fluconazole as antifungal prophylaxis in alloHCT recipients post-transplant, a result largely driven by fluconazole’s lack of anti-mould activity; this finding is consistent with a previously published meta-analysis [8]. Among the mould-active azoles, posaconazole and voriconazole reduced the risk of IA more than itraconazole. In contrast, itraconazole was the most effective in preventing IC, which is also consistent with published meta-analyses [20,44]. Compared with fluconazole, voriconazole had the greatest probability of reducing OLAT use, which may have both clinical and pharmacoeconomic implications. Other outcomes of potential interest, such as incidence of possible IFI and fungal-free survival, could not be evaluated, since relevant data were not consistently reported in the eligible RCTs.

Currently, IFI caused by *Aspergillus* spp. predominate [2,3] and reduced intensity conditioning (RIC) transplants are now commonplace, with 42% of patients in the recent voriconazole versus itraconazole study having undergone RIC/nonmyeloablative conditioning [10]. In RIC patients, IFIs, particularly invasive mould infections, tend to occur during the late post-engraftment period (ie, after day 100) [2,45]; these patients may therefore require longer periods of mould-active antifungal prophylaxis. Of note, the studies included into our evidence network assessed patients for a post-engraftment period of up to 180 days, but the at-risk period for invasive mould infections extends beyond this period [2,3,45].

The included studies were heterogeneous in terms of the study design, patient population and risk of IFI, such that recognition of possible treatment effects may have been obscured. For example, the IFI rates varied across the studies and are likely to reflect a similar variance in IFI risk. The highest IFI rate was observed in the study by Winston and colleagues [29] where a greater proportion of fluconazole recipients received unrelated donor stem cells, and had a higher incidence of acute and chronic graft-versus-host disease (GvHD), thus amplifying the difference in IFI event rates between study and control groups.

The incidence of grades II–IV acute GvHD as a risk factor for IFI also varied significantly among the studies from 100% [27], to 64% [26], 46% [10], 41% [28], and 37% [29]. The IFI risk may have been influenced by the heterogeneity of conditioning regimen intensities among the studies included in the analysis.

All (100%) subjects in the Seattle study [26] and the study of Blood and Marrow Clinical Trials Network [28] received myeloablative conditioning regimens compared to 78% in the study by Winston et al. [29] and 58% in the multicentre European study [10]. Accordingly, the influence of pre-engraftment myelosuppression and cytotoxic therapy-induced intestinal epithelial damage on IFI risk may have varied among the studies.

There was a significant variance in the prophylaxis start dates among the studies ranging from the beginning of conditioning [26], to the day of transplant [10,28], the day after transplant [29], and the day of documentation of GvHD (median of day 64 post-transplant) [27]. This latter study only reported IFI incidence at 112 days post-treatment initiation, and did not address the incidence of IFI and OLAT use between the time of transplant and the start of study prophylaxis [27]. The decision to include the posaconazole trial was validated by the alignment of the results of the base-case analysis with those of the post-hoc sensitivity analysis in which the study was excluded.

Similarly, there were significant variations in toxicity- or intolerance-driven drug withdrawal rates that likely influenced prophylaxis drug exposure and efficacy. Itraconazole withdrawal rates ranged from a low of 8.5% [29], to 36% [26] and 43% [10]. Fluconazole withdrawal rates ranged from 1.5% [29], to 16% [26], 38% [27], and 44% [28]. Voriconazole withdrawal rates were similar at 41% [28] and 37% [10]. The posaconazole withdrawal rate was 34% [27]. Our inability to control for all of these different variables, reflected in the heterogeneity of the included studies, reduced the sensitivity of the analysis to detect treatment effects for the outcome of interest, the use of a random effects model notwithstanding.

Application of the 2008 revised definitions for the end-points for the prophylaxis studies [46] may have provided a more robust basis for prophylaxis efficacy outcomes as has been noted for treatment outcomes [47,48]; however, we used the definitions for invasive fungal infection employed in the methods of the included trials for consistency.

Comparative efficacy notwithstanding, considerations that may impact the decision-making process include cost differences, ease of use, availability of an intravenous formulation (in case of mucositis and/or intestinal GvHD), adverse event and drug-drug interaction profile, availability of expertise and diagnostic tools for early diagnosis of invasive mould infection, and local IFI epidemiology. While substantial cost differences exist between generic fluconazole and itraconazole on the one hand and posaconazole and voriconazole on the other, the drug costs associated with OLAT should be considered as well. Oral and gastrointestinal mucositis may limit the role of posaconazole due to the unavailability of an intravenous formulation and the requirement for administration with a full meal [49]. Of note, all of the mould-active azoles adversely interact with immunomodulatory and antineoplastic drugs. Prophylactic fluconazole may be a worthwhile alternative to mould-active prophylaxis in alloHCT in centres practicing early diagnostics-driven therapy [8].

It should be noted that patient-level data (from published papers), rather than (raw) data extracted from clinical study reports, were used to drive this MTC meta-analysis.

## Conclusions

We cannot firmly conclude that specific agents performed better than others in any of the evaluated outcomes, due to the width of credible intervals in the base-case analysis. However, from a medical decision-making point of view, the results support the selection of mould-active azoles over fluconazole for the prevention of IFIs in alloHCT recipients post-transplant. In addition, posaconazole and voriconazole may be preferable for protection from IA, itraconazole for protection from IC, and voriconazole for reducing OLAT use, based on the respective relatively high posterior probabilities (which were nevertheless <95% in most cases). In order to more conclusively assess comparative efficacy, future research in the form of additional (multi-arm) RCTs is likely to be necessary. Given the current paucity of comparative data between the mould-active azoles, other considerations such as availability of an intravenous formulation, local IFI epidemiology, and drug costs, may be factors to consider when choosing between these agents.

## Additional file

Additional file 1:
**Systematic review and mixed treatment comparison of randomized clinical trials of primary antifungal prophylaxis in allogeneic hematopoietic cell transplant recipients.**
