# Supplementary material for: Systematic review and mixed treatment comparison meta-analysis of randomized clinical trials of primary oral antifungal prophylaxis in allogeneic hematopoietic cell transplant recipients
Source: BMC Infect Dis. 2015 Mar 17;15:128. doi: 10.1186/s12879-015-0855-6 (PMC4374298; doi:10.1186/s12879-015-0855-6)
Supplement: Additional file 1: — Systematic review and mixed treatment comparison of randomized clinical trials of primary antifungal prophylaxis in allogeneic hematopoietic cell transplant recipients. [file 12879_2015_855_MOESM1_ESM.docx]

ADDITIONAL FILES

**Systematic review and mixed treatment comparison of randomized clinical trials of primary antifungal prophylaxis in allogeneic hematopoietic cell transplant recipients**

EJ Bow,^1^ DJ Vanness,^2^ M Slavin,^3^ C Cordonnier,^4^ OA Cornely,^5^ D. I. Marks,^6^ A Pagliuca,^7^ C Solano,^8^ L Cragin,^9^ AJ Shaul,^9^ S Sorensen,^9^ R. Chambers,^10^ M Kantecki,^11^ D Weinstein,^11^ and H Schlamm^12^

^1^CancerCare Manitoba, Winnipeg, Canada; ^2^University of Wisconsin and Visiting Scientist at Evidera, Madison, USA; ^3^Royal Melbourne Hospital, Melbourne, Australia; ^4^Assistance Publique-Hopitaux de Paris, Hôpital Henri Mondor and Université Paris-Est-Créteil, Creteil, France; ^5^Department I of Internal Medicine, Clinical Trials Centre Cologne, ZKS Köln, BMBF 01KN1106, Center for Integrated Oncology CIO KölnBonn, Cologne Excellence Cluster on Cellular Stress Responses in Aging-Associated Diseases (CECAD), University of Cologne, Cologne, Germany; ^6^University Hospitals Bristol NHS Foundation Trust, Bristol, UK; ^7^King's College Hospital, London, UK; ^8^Hospital Clínico, INCLIVA Foundation, University of Valencia, Spain; ^9^Evidera, Bethesda, USA; ^10^Pfizer, Collegeville, USA; ^11^Pfizer, Paris, France; ^12^HTS Pharma Consulting, New York, USA

Table of Contents

[Methods – Detailed description and methodology of mixed-treatment comparison 2](#_Toc367271235)

[Results – Flow chart of systematic literature review 16](#_Toc367271236)

[Results – Key information about each identified RCT 17](#_Toc367271237)

[Results – Data extracted from each RCT 21](#_Toc367271238)

[Results – Overall estimates of heterogeneity 22](#_Toc367271239)

[Results – Sensitivity analysis excluding the single posaconazole trial 23](#_Toc367271240)

[References 24](#_Toc367271241)

# Methods – Detailed description and methodology of mixed-treatment comparison

In the current analysis, a mixed treatment comparison (MTC) was used to obtain estimates for infection rates, overall survival, and use of other licensed antifungal therapy (OLAT) [1]. This method of evidence synthesis is increasingly being used in health technology appraisals throughout the world [2]. Taking infection rates for each treatment directly from clinical trials is problematic because of the differences in trial populations, designs and other clinical factors that would cause the “baseline” rates of infection, overall survival, and OLAT use to vary. Failing to account for differences in baseline factors would cause inaccurate estimates of infection rates, overall survival, and OLAT use between treatments. These differences in treatment effectiveness have substantial clinical relevance and are also most likely to drive results in an incremental cost-effectiveness analysis.

In an MTC, statistical models need to be specified for two things. Firstly, the baseline rate of infection, overall survival, or OLAT use needs to be modeled. Placebo or minimal care is often chosen as the baseline treatment – ie, what would the rate of infection, overall survival, or OLAT use be in each trial if there were no active treatment? For practical reasons, baseline treatment is often chosen as the common comparator. For our analysis, this was fluconazole (FLU), not placebo or minimal care. We modeled baseline treatment for each trial even if the trial itself had no arm using the baseline treatment. For example, we could predict what infection rates and OLAT use would have been observed with FLU, if FLU had been a treatment arm in the IMPROVIT study [3].

Due to differences in patient characteristics and supportive medical care, we would not expect ex ante to see infection rates, overall survival, or OLAT use with FLU treatment in the IMPROVIT study equivalent to those observed by Winston *et al* in 2003 [4]. For this reason, the baseline rates of infection, overall survival, and OLAT use were modeled using the unconstrained baseline assumption. By completely separating the baseline parameters for each trial, the unconstrained baseline assumption provides maximum flexibility. All else equal, estimating more parameters in this type of statistical model decreases the precision of inference (resulting in wider confidence intervals, or in the case of Bayesian inference, wider credible intervals – discussed below). If we had strong beliefs that the baseline rates would be the same or similar in each trial, we could use either a single (fixed effect) baseline or a constrained (random effect) baseline, respectively, to improve our precision. However, improving precision comes at the cost of increasing potential bias, and upon consultation with the clinical experts it was decided that the conservative approach was warranted.

The second (and ultimately most important) item we need to model statistically is the set of all possible treatment effects. A treatment effect is a measure of the difference in infection rates, overall survival, or OLAT use between any two treatments (eg, FLU vs itraconazole (ITR), FLU vs voriconazole (VOR), FLU vs posaconazole (POS), ITR vs VOR, ITR vs POS, VOR vs POS). For technical reasons, we often use mathematical transformations of the difference (such as the rate ratio, odds-ratio or log-odds-ratio), but it is always possible to move back to the actual difference. In our analysis, we modeled the log-odds-ratio of infection rates, overall survival, or OLAT use. If the log-odds-ratio of infection between two treatments (B relative to A) is zero, then the infection rates under each treatment are equal. For example, irrespective of whether the infection rates were 1%, 5% or 99%, they would all give a log-odds-ratio of 0. A log-odds-ratio of one implies that the odds of infection under treatment B is about 2.7 (ie, 2.7 = e ^1) times the odds of infection under treatment A, while a log-odds-ratio of –1 implies that the odds of infection under treatment B is 1/2.7 or 0.37 (ie, 0.37 = e^-1) times the odds of infection under treatment A. Again, there are any number of different rates of infection for A and B which give the same log-odds-ratio.

In our analysis with four treatments, ie, FLU, POS, ITR and VOR, there are six possible pairwise comparisons (eg, FLU vs ITR, FLU vs VOR, FLU vs POS, ITR vs VOR, ITR vs POS, VOR vs POS). The three comparisons of treatments with baseline (FLU), ie, POS vs FLU, ITR vs FLU and VOR vs FLU are called “basic” comparisons. Our model uses one parameter to estimate each of these basic comparisons, plus one additional parameter to account for potential heterogeneity in estimates of basic treatment effect between studies. This parameter means that we do not require that every trial providing evidence about a basic comparison is estimating exactly the same (fixed) treatment effect. For example, we acknowledge that the estimates of ITR vs FLU in Marr *et al,* 2004 [5] and Winston *et al*, 2003 [4] differ from one another not just because of sampling variability, but also because of differences in study designs and populations. Specifically, we say that the observed treatment effect in a trial differs from the “true” treatment effect by a normally-distributed error term with mean zero and unknown variance. The unknown variance is estimated from the data itself. If many trials with the same basic comparison have widely different results, then the variance (heterogeneity) will be high. If all trial treatment effect estimates are close to one another, then the variance (heterogeneity) will be low. Note that we assume the heterogeneity is the same for all basic comparisons (ie, ITR vs FLU trial results have as much variability as VOR vs FLU or POS vs FLU trial results). Because we do not have more than one trial for POS vs FLU or VOR vs FLU, we cannot estimate heterogeneity parameters for each type of basic comparison, and our assumption of equal variability across comparisons cannot be tested. These assumptions about the type of heterogeneity come under the category of exchangeability. To satisfy exchangeability, there should be no a priori ability of the analyst to rank-order trials by their predicted treatment effect (ie, relative rates of infection, overall survival, or OLAT use between two treatments) based on characteristics of the trial design and population alone.

The three remaining comparisons are called “functional” comparisons: VOR vs POS, ITR vs POS and ITR vs VOR, because they can be estimated as functions of the basic comparisons. For example, VOR vs POS can be obtained indirectly as a function of POS vs FLU and VOR vs FLU. Specifically, the log-odds-ratio has the convenient property that the log-odds-ratio of VOR vs POS equals the log-odds-ratio of VOR vs FLU minus the log-odds-ratio of POS vs FLU. We do not use any additional parameters to estimate the functional comparisons, since they are entirely determined by the basic comparisons. In many instances, the functional comparisons are actually the objects of interest because they represent head-to-head comparisons of active treatments.

By assuming that head-to-head comparisons can be derived indirectly, an MTC model allows both head-to-head and baseline comparator trials to contribute evidence. For example, the treatment effect of ITR compared with VOR is informed not only by the head-to-head IMPROVIT trial, but also by the Marr *et al*, 2004 [5] and Winston *et al*, 2003 [4] trials of ITR vs FLU and the Wingard *et al*, 2010 [6] trial of VOR vs FLU. Furthermore, even though POS has never been directly compared to ITR or VOR in a head-to-head trial, treatment effects can still be estimated because each of those treatments has been previously compared with FLU. The major assumption being made here is called the consistency assumption [7]. One way to think of this assumption is to consider the treatment effect estimate of ITR relative to VOR from the IMPROVIT trial. Imagine that the Wingard *et al*, 2010 [6] study of VOR vs FLU also included a treatment arm whereby patients were given ITR. Consistency requires that the log-odds-ratio of infection rates, overall survival, or OLAT use of VOR relative to ITR in IMPROVIT would not be expected a priori to be substantially different than the log-odds-ratio of VOR relative to ITR that would have been observed if the Wingard *et al*, 2010 [6] study had also included an ITR arm. Note that this assumption does not require that the rates of infection be the same, but rather that the relative rates of infection are similar. Another way to think of this is to imagine that all trials could have included all four treatments of interest, but that the data for one or more arms in each trial is “missing” (eg, data for POS and FLU are missing from the IMPROVIT study). If investigators could predict ex ante which arms would be missing from each trial given the study population and trial design, then there would be an a priori reason to suspect that the data are inconsistent.

The more trials that are available, the easier it is to check for patterns in the results that suggest violations of our assumptions of exchangeability and consistency. Unfortunately, in our analysis, we have only one study (IMPROVIT) that estimates a head-to-head comparison. And, we only have one basic comparison (ITR vs FLU) for which there is more than one trial [4, 5]. Therefore, we rely heavily on untestable assumptions and must at the very least not have a priori reasons to reject these assumptions. The trial populations informing the MTC analysis were heterogeneous, eg, all patients in the RCT conducted by Ullman *et al*, 2007 [8] had graft versus host disease (GVHD) whereas those in the RCT by Marks *et al,* 2011 [3] included patients with and without GVHD. The study designs were also heterogeneous, eg, prophylaxis was initiated at the time of allogeneic hematopoietic stem cell transplantation (alloHCT) in Marks *et al*, 2011 [3], whereas in the RCT by Ullman *et al,* 2007 [8] prophylaxis was not initiated until GVHD developed after alloHCT. However, despite the acknowledged heterogeneity, there were no a priori reasons to reject the assumptions of exchangeability and consistency.

In theory, MTC models can be estimated using classical statistical methods such as maximum likelihood. However, the dominant method of estimation is Bayesian. In Bayesian analysis, unknown parameters of interest are treated as random variables. As random variables, they have a probability distribution that summarizes our knowledge about the unknown parameter. The distribution of a parameter before observing data is called a prior. Priors with large variances mean that the analyst has relatively little information about the parameters before observing the dataset to be analyzed. Priors with small variances mean that the analyst already has prior information, perhaps from outside data or expert opinion. The prior distribution is combined with a statistical likelihood function and Bayes’ Rule to produce a posterior distribution, which summarizes our knowledge about the parameter after observing the data.

The raw results of our MTC analysis are posterior distributions for nine parameters: five study baseline parameters (the predicted rate probability of infection, overall survival, or OLAT use on FLU for each of the included studies); three basic comparison parameters (the log-odds-ratio of infection, overall survival, or OLAT use for POS vs FLU, VOR vs FLU and ITR vs FLU); and one heterogeneity parameter (variability of study treatment effects relative to the true treatment effect; likely for reasons beyond sampling variability). Posterior distributions for the three “functional” (direct) comparisons (VOR vs POS, ITR vs POS and ITR vs VOR) can be calculated from the posteriors of the basic comparisons.

The posterior distributions are then translated from the log-odds-ratio scale into estimates of infection rates, overall survival, and OLAT use for each treatment. The estimated probabilities can then be compared to help inform clinical decision-making, and, in addition, used as clinical inputs in a cost-effectiveness analysis. However, in order to do this, estimates of both the baseline (FLU) rate of infection and the three basic comparison estimates (POS vs FLU, VOR vs FLU and ITR vs FLU) are required. As demonstrated above, the comparison estimates alone are not enough because there are many different pairs of event rates that produce the same log-odds-ratio. Finding the appropriate baseline event rate can, therefore, be challenging. From the model itself, we have five different estimates of infection rates, overall survival, or OLAT use on FLU, one for each trial. Typically, these rates are just averaged over all trials that included an arm for the baseline treatment (in our analysis, there are four). However, our results suggest a strong time trend in baseline infection rates. Therefore, we decided to use the estimated baseline event rates for the Wingard *et al*, 2010 [6] study only, since it was the most recent trial including a FLU arm, and its population is similar to our target population of interest for the cost-effectiveness analysis.

We used simple mathematical formulae to transform the log-odds of the rates of baseline infection, overall survival, or OLAT use back into estimates of the actual probability of infection, overall survival, or OLAT use under each of the four treatments. The result is not a single set of four point estimates, but rather four posterior distributions summarizing our knowledge about the infection, overall survival, or OLAT use rates. To avoid confusion, note that each different outcome (invasive aspergillosis, invasive candidiasis, other invasive fungal infections [IFI], overall survival, and OLAT) is estimated using a separate model; as such, there are posterior distributions for each of four outcomes for each of four treatments (ie, 4 x 4 = 16 posterior distributions). To summarize each posterior distribution, we need to pick a statistic such as the mean or median. Because each posterior distribution in our analysis is skewed, we felt that the posterior median was the best overall estimate of the event rate to summarize the results of the MTC analysis, and to use as a point estimate in the base case cost-effectiveness analysis. The rationale for this is similar as to why median survival is often used as a measure of treatment effectiveness, rather than mean survival, when there are outliers present in the data (when outliers are absent in the data, the mean and median are very “close” in value; when outliers are present, the median and mean become dissimilar). In the cost-effectiveness analysis, we used the entire posterior distribution to conduct probabilistic sensitivity analysis. This type of analysis is meant to show the overall uncertainty about the estimated cost-effectiveness ratios, given uncertainty about input parameters. Because the posterior itself is the best measure of uncertainty about infection, overall survival, or OLAT use rates, we make direct use of the posteriors as described below.

In the initial version of the model, noninformative priors for both types of parameters (baseline and treatment effect) were specified using a normal distribution with a mean of zero and a variance of 1000. For the baseline, since we are operating on the log-odds scale, this represents a range of event rates from infinitesimally close to zero (roughly 1e–25) to infinitesimally close to one (1–1e-25). For the relative effects, this allows extraordinarily high reductions or increases in event rates, ie, roughly +/– 25 orders of magnitude. In the presence of informative data, uninformative priors are “swamped” by the data, and extreme event rates and treatment effects are ruled out. However, with the relatively small amount of data being combined, using unbounded noninformative priors still allows for relatively extreme values and essentially impossible estimates of event rates under each treatment.

Model code for the MTC using a noninformative prior

model{

for(i in 1:N_ARMS){

INFECTIONS[i] ~ dbin(p[i],N_PATIENTS[i])

logit(p[i])<-min(max(mu[STUDY[i]] + delta[i]*(1-equals(TREATMENT[i],CONTROL[i])),-12),12)

delta[i] ~ dnorm(mu.d[i],prec.d)

mu.d[i] <- d[TREATMENT[i]]-d[CONTROL[i]]

rhat[i] <- p[i] * N_PATIENTS[i] # predicted r for each arm

eps[i] <- (INFECTIONS[i]-rhat[i])/max(.00001,pow(N_PATIENTS[i]*p[i]*(1-p[i]),.5)) # standardized level 1 residuals

}

#Unconstrained baseline event rates

for(j in 1:N_STUDIES){

mu[j] ~ dnorm(0,.01)

mu.1[j] <- equals(CONTROL[2*j],1)*mu[j] #Note this form of code only works when all trials are 2 arm

logit(p.mu[j]) <- min(max(mu[j],-12),12)

}

mubar <-sum(mu.1[])/N_NONFLUCONTROL #Calculate average for FLU baseline trials only

#mubar <- mu.1[1] #Use Marr 2004 as baseline IA estimate

#Give priors for log-odds-ratios

d[1]<-0

for (k in 2:N_TREATMENTS){d[k] ~ dnorm(0,.01)}

#Prior for RE precision

prec.d <- 1/pow(sd.d,2)

sd.d ~ dnorm(0,.01)I(0,)

#Calculate treatment effects, T[k], on natural scale

#for (k in 1:N_TREATMENTS){logit(T[k]) <- mubar + d[k]}

#Rank the treatment effects (with 1=best) & record the best treatment

for(k in 1:N_TREATMENTS){

rk[k]<- rank(d[],k)

best[k]<-equals(rk[k],1)

}

#Better than FLU

for(k in 2:N_TREATMENTS){

btf[k-1]<-1-step(d[k])

}

abtf <- 1-equals(rk[1],1)

for(k in 1:N_TREATMENTS){or.d[k] <- exp(d[k])}

#All pairwise log-odds-ratios, odds-ratios and relative risks

for (c in 1:(N_TREATMENTS-1)){

for (k in (c+1):N_TREATMENTS){

lor[c,k] <- d[k] - d[c]

log(or[c,k]) <- lor[c,k]

}

}

}

Two different types of sensitivity analyses were conducted in regards to the MTC analyses for probability of IFI/IA/IC, overall survival, OLAT and mortality. First, we examined the sensitivity to inclusion of a priori heterogeneous studies (Ullman 2007 [8] for all end points and Marr 2004 [5] for mortality). Second, we examined sensitivity to the “prior” distribution on the heterogeneity parameter. When conducting mixed or indirect treatment comparisons using a random-effects model, we assumed that the treatment effects (difference in effects between treatments) are random variables – ie, they come from distributions with a mean equal to the true treatment effect and an unknown variance. The variance is unknown because we do not know for sure how treatment effects may vary from study to study as a result of variations in design, population, etc. We simply know that the effects are likely to vary. In classical random-effects meta-analysis, a “heterogeneity parameter” representing the treatment effect variance between studies is estimated from the data and is treated as known. In Bayesian random-effects meta-analysis, the heterogeneity parameter is also estimated from the data, but we admit that our estimate of the variance has some uncertainty because the variance is being estimated from a sample of possible studies. Therefore, we have to assign a prior to this parameter. In standard “noninformative prior” Bayesian meta-analysis (which is almost always the base case for published MTCs) we place priors indicating that we know nothing about the unknown parameters (including the heterogeneity parameter). When only a small number of studies are included, the ability to estimate the heterogeneity parameter becomes very limited, and consequently, the “posterior” ends up looking a lot like the “prior.” Usually, this means that relatively extreme amounts of variance between studies (ie, ratios of odds-ratios between studies of hundreds or even thousands) cannot be ruled out. This uncertainty propagates through to the estimates of the treatment effects themselves, and is largely why many of the 95% credible intervals in our base case analyses include outlandish values, even though the interquartile ranges are relatively stable. Such analyses may be extremely sensitive to variations in the prior distribution on the heterogeneity parameter [9].

For the sensitivity analysis, we used the “empirical Bayes” method described by DuMouchel and Normand in Stangl and Berry [10] and which has been shown to perform reasonably well by Lambert *et al* [9]. Empirical Bayes methods derive priors for “nuisance parameters” from the data itself. This specific method uses the calculated standard errors from each trial’s estimated treatment effect to estimate the parameter of a log-logistic prior for the heterogeneity parameter. Empirical Bayesian methods provide a nice bridge between Bayesian and Classical meta-analysis and were deemed acceptable for sensitivity analysis purposes.

Model code for the MTC using an empirical prior

model{

for(i in 1:N_ARMS){

INFECTIONS[i] ~ dbin(p[i],N_PATIENTS[i])

logit(p[i])<-min(max(mu[STUDY[i]] + delta[i]*(1-equals(TREATMENT[i],CONTROL[i])),-12),12)

delta[i] ~ dnorm(mu.d[i],prec.d)

mu.d[i] <- d[TREATMENT[i]]-d[CONTROL[i]]

rhat[i] <- p[i] * N_PATIENTS[i] # predicted r for each arm

eps[i] <- (INFECTIONS[i]-rhat[i])/max(.00001,pow(N_PATIENTS[i]*p[i]*(1-p[i]),.5)) # standardized level 1 residuals

}

#Unconstrained baseline event rates

for(j in 1:N_STUDIES){

mu[j] ~ dnorm(0,.01)

mu.1[j] <- equals(CONTROL[2*j],1)*mu[j] #Note this form of code only works when all trials are 2 arm

logit(p.mu[j]) <- min(max(mu[j],-12),12)

}

mubar <-sum(mu.1[])/N_NONFLUCONTROL #Calculate average for FLU baseline trials only

#mubar <- mu.1[1] #Use Marr 2004 as baseline IA estimate

#Give priors for log-odds-ratios

d[1]<-0

for (k in 2:N_TREATMENTS){d[k] ~ dnorm(0,.01)}

#Prior for RE precision

prec.d <- 1/pow(sd.d,2)

p.d ~ dunif(0,1)

sd.d <- p.d*S0/(1-p.d)

#Calculate treatment effects, T[k], on natural scale

#for (k in 1:N_TREATMENTS){logit(T[k]) <- mubar + d[k]}

#Rank the treatment effects (with 1=best) & record the best treatment

for(k in 1:N_TREATMENTS){

rk[k]<- rank(d[],k)

best[k]<-equals(rk[k],1)

}

#Better than FLU

for(k in 2:N_TREATMENTS){

btf[k-1]<-1-step(d[k])

}

abtf <- 1-equals(rk[1],1)

for(k in 1:N_TREATMENTS){or.d[k] <- exp(d[k])}

#All pairwise log-odds-ratios, odds-ratios and relative risks

for (c in 1:(N_TREATMENTS-1)){

for (k in (c+1):N_TREATMENTS){

lor[c,k] <- d[k] - d[c]

log(or[c,k]) <- lor[c,k]

}

}

}

# Results – PRISMA flow chart for systematic literature review

CENTRAL, Cochrane Central Register of Controlled Trials; CSR, Clinical Study Report; PRISMA, Preferred Reporting Items for Systematic Reviews and Meta-Analyses; RCT, randomized controlled trial.

# Results – Key information about each identified RCT

**Winston 2003 [4]**

- Treatments: Itraconazole (200 mg iv every 12 hours for 2 days then 200 mg/day iv or 200 mg oral solution every 12 hours) vs fluconazole (400 mg/day iv or oral) for 100 days
- Study design: Multicenter, open-label, superiority
- Primary end point: Incidence of invasive fungal infection
- Study population: Allogeneic HCT patients (≥13 years)
- Study size: 140 randomized (itraconazole, n = 72; fluconazole, n = 68), 138 analyzed (itraconazole, n = 71; fluconazole, n = 67)
- Median follow-up: [not stated]

**Marr 2004 [5]**

- Treatments: Itraconazole (2.5 mg/kg oral solution 3 times daily, or 200 mg iv daily) vs fluconazole (400 mg/day oral or iv) for 120–180 days
- Study design: Single site, open-label, superiority
- Primary end point: Incidence of proven or probable fungal infection
- Study population: Allogeneic HCT patients (≥13 years)
- Study size: 304 randomized (itraconazole, n = 153; fluconazole, n = 151), 299 analyzed (itraconazole, n = 151; fluconazole, n = 148)
- Median follow-up: itraconazole, 23.6 months; fluconazole, 23.3 months

**Ullmann 2007[8]**

- Treatments: Posaconazole (200 mg oral suspension 3 times daily) vs fluconazole (400 mg oral once daily) for 112 days
- Study design: Multicenter, double-blind, noninferiority and superiority
- Primary end point: Incidence of proven or probable invasive fungal infections (from randomization to day 112 of the fixed treatment period of the study)
- Study population: Allogeneic HCT patients with acute GVHD, grade II to IV or chronic extensive GVHD, or receiving intensive immunosuppressive therapy (≥13 years)
- Study size: 600 randomized (posaconazole, n = 301; fluconazole, n = 299), 600 analyzed (posaconazole, n = 301; fluconazole, n = 299)
- Median follow-up: [not stated]

**Wingard 2010 [6]**

- Treatments: Voriconazole (200 mg oral or iv twice-daily) vs fluconazole (400 mg/day oral or iv) for 100 days and up to 180 days (in higher risk patients)
- Study design: Multicenter, double-blind, superiority
- Primary end point: Fungal-free survival (alive and free from proven, probable or presumptive IFI) at 180 days post-transplant
- Study population: Myeloablative allogeneic HCT patients (≥2 years)
- Study size: 600 randomized (voriconazole, n = 305; fluconazole, n = 295), 600 analyzed (voriconazole, n = 305; fluconazole, n = 295)
- Median follow-up: [not stated]

**Marks 2011 [3]**

- Treatments: Voriconazole (6 mg/kg iv twice-daily then 200 mg oral twice-daily for patients >40 kg and 100 mg oral twice-daily for patients <40 kg) vs itraconazole (200 mg iv twice-daily then 200 mg oral twice-daily) for at least 100 days and up to 180 days
- Study design: Multicenter, open-label, superiority
- Primary end point: Success of antifungal prophylaxis at day 180
- Study population: Myeloablative or reduced intensity allogeneic HCT patients (≥12 years)
- Study size: 503 randomized (voriconazole, n = 243; itraconazole, n = 260), 465 analyzed (voriconazole, n = 224; itraconazole, n = 241)
- Median follow-up: [not stated]

# Results – Data extracted from each RCT

For each outcome, the numbers of patients with the respective event out of the overall study population are provided, along with the corresponding rate.

| **Study** | **Incidence of proven/probable invasive fungal infection** | **Incidence of proven/probable invasive aspergillosis** | **Incidence of proven/probable invasive candidiasis** | **Proportion of patients who received other licensed antifungal therapy** | **All-cause mortality** |
| --- | --- | --- | --- | --- | --- |
| **Winston 2003** | FLU: 17/67 (25.4%)  ITR: 6/71 (8.5%) | FLU: 8/67 (11.9%)  ITR: 3/71 (4.2%) | FLU: 8/67 (11.9%)  ITR: 2/71 (2.8%) | FLU: (N/A)  ITR: (N/A) | FLU: 28/67 (41.8%)  ITR: 32/71 (45.1%) |
| **Marr 2004** | FLU: 25/148 (16.9%)  ITR: 19/151 (12.6%) | FLU: 20/148 (13.5%)  ITR: 16/151 (10.6%) | FLU: 5/148 (3.4%)  ITR: 4/151 (2.6%) | FLU: 25/148 (16.9%)  ITR: 19/151 (12.6%) | FLU: 44/148 (29.7%)  ITR: 55/151 (36.4%) |
| **Ullmann 2007** | FLU: 27/299 (9.0%)  POS: 16/301 (5.3%) | FLU: 21/299 (7.0%)  POS: 7/301 (2.3%) | FLU: 4/299 (1.3%)  POS: 4/301 (1.3%) | FLU: 29/288 (10.1%)  POS: 31/291 (10.7%) | FLU: 59/299 (19.7%)  POS: 58/301 (19.3%) |
| **Wingard 2010** | FLU: 24/295 (8.1%)  VOR: 14/305 (4.6%) | FLU: 17/295 (5.8%)  VOR: 9/305 (3.0%) | FLU: 5/295 (1.7%)  VOR: 3/305 (1.0%) | FLU: 89/295 (30.2%)  VOR: 73/305 (23.9%) | FLU: 59/295 (20.0%)  VOR: 57/305 (18.7%) |
| **Marks 2011** | ITR: 5/241 (2.1%)  VOR: 3/224 (1.3%) | ITR: 5/241 (2.1%)  VOR: 1/224 (0.4%) | ITR: 0/241 (0.0%)  VOR: 2/224 (0.9%) | ITR: 101/241 (41.9%)  VOR: 67/224 (29.9%) | ITR: 44/241 (18.3%)  VOR: 40/224 (17.9%) |

Abbreviations: FLU, fluconazole; ITR, itraconazole; POS, posaconazole; VOR, voriconazole.

# Results – Overall estimates of heterogeneity

The table below provides estimates of heterogeneity, ie, the posterior 50th percentile of the heterogeneity parameter expressed as log-odds.

| **Outcome** | **Base case MTC**  **(noninformative prior)** | **Sensitivity analysis MTC**  **(empirical prior)** |
| --- | --- | --- |
| **Proven/probable invasive fungal infection** | 0.813 | 0.231 |
| **Proven/probable invasive aspergillosis** | 1.048 | 0.260 |
| **Proven/probable invasive candidiasis** | 1.872 | 0.500 |
| **Other licensed antifungal therapy** | 1.201 | 0.162 |
| **Mortality** | 0.301 | 0.099 |

# Results – Sensitivity analysis excluding the single posaconazole trial

| **Comparator** | **Posterior odds-ratio relative to fluconazole**  **(interquartile range)^a^** | **Posterior probability of having lower incidence than fluconazole (%)** | **Posterior probability of having the lowest incidence of all treatments (%)** |
| --- | --- | --- | --- |
| **All-cause mortality** | | | |
| Fluconazole  Itraconazole  Voriconazole | –  1.17 (0.96–1.43)  1.02 (0.82–1.27) | –  29  48 | 42  17  41 |
| **Proven/probable IFI at 180 days** | | | |
| Fluconazole  Itraconazole  Voriconazole | –  0.52 (0.34–0.78)  0.46 (0.27–0.74) | –  84  84 | 7  40  54 |
| **Proven/probable IA at 180 days** | | | |
| Fluconazole Itraconazole  Voriconazole | –  0.69 (0.41–1.15)  0.33 (0.16–0.60) | –  70  86 | 8  20  73 |
| **Proven IC at 180 days** | | | |
| Fluconazole  Itraconazole  Voriconazole | –  0.27 (0.10–0.60)  1.17 (0.42–4.43) | –  84  46 | 11  72  16 |
| **OLAT use at 180 days** | | | |
| Fluconazole  Itraconazole  Voriconazole | –  0.91 (0.47–1.66)  0.63 (0.34–1.14) | –  55  72 | 19  24  57 |

^a^ Estimates less than zero indicate a reduced probability of proven/probable IFI at 180 days relative to fluconazole.

#

# References

1. Lu G, Ades AE: **Combination of direct and indirect evidence in mixed treatment comparisons**. *Stat Med* 2004, **23**:3105-3124.

2. Sutton A, Ades AE, Cooper N, Abrams K: **Use of indirect and mixed treatment comparisons for technology assessment.** *Pharmacoeconomics* 2008, **26**:753-767.

3. Marks DI, Pagliuca A, Kibbler CC, Glasmacher A, Heussel CP, Kantecki M, Miller PJ, Ribaud P, Schlamm HT, Solano C, Cook G; IMPROVIT Study Group*:* **Voriconazole versus itraconazole for antifungal prophylaxis following allogeneic haematopoietic stem-cell transplantation**. *Br J Haematol* 2011, **155**:318-327.

4. Winston DJ, Maziarz RT, Chandrasekar PH, Lazarus HM, Goldman M, Blumer JL, Leitz GJ, Territo MC: **Intravenous and oral itraconazole versus intravenous and oral fluconazole for long-term antifungal prophylaxis in allogeneic hematopoietic stem-cell transplant recipients. A multicenter, randomized trial**. *Ann Intern Med* 2003, **138**:705-713.

5. Marr KA, Crippa F, Leisenring W, Hoyle M. Boeckh M, Balajee SA, Nichols WG, Musher B, Corey L: **Itraconazole versus fluconazole for prevention of fungal infections in patients receiving allogeneic stem cell transplants**. *Blood* 2004, **103**:1527-1533.

6. Wingard JR, Carter SL, Walsh TJ, Kurtzberg J, Small TN, Baden LR, Gersten ID, Mendizabal AM, Leather HL, Confer DL, Maziarz RT, Stadtmauer EA, Bolaños-Meade J, Brown J, Dipersio JF, Boeckh M, Marr KA: **Randomized, double-blind trial of fluconazole versus voriconazole for prevention of invasive fungal infection after allogeneic hematopoietic cell transplantation**. *Blood* 2010, **116**:5111-5118.

7. Cooper NJ, Sutton AJ, Morris D, Ades AE, Welton NJ: **Addressing between-study heterogeneity and inconsistency in mixed treatment comparisons: Application to stroke prevention treatments in individuals with non-rheumatic atrial fibrillation**. *Stat Med* 2009, **28**:1861-1881.

8. Ullmann AJ, Lipton JH, Vesole DH, Chandrasekar P, Langston A, Tarantolo SR, Greinix H, Morais de Azevedo W, Reddy V, Boparai N, Pedicone L, Patino H, Durrant S. **Posaconazole or fluconazole for prophylaxis in severe graft-versus-host disease**. *N Engl J Med* 2007, **356**:335-347.

9. Lambert PC, Sutton AJ, Burton PR, Abrams KR, Jones DR: **How vague is vague? A simulation study of the impact of the use of vague prior distributions in MCMC using WinBUGS**. Stat Med 2005, **24**:2401-2428.

10. DuMouchel W, Normand S-L: *Computer-modeling and graphical strategies for meta-analysis*. In: Stangl DK, Berry DA, editors. Meta-analysis in Medicine and Health Policy. New York, NY: Marcel Dekker, Inc., 2000:108-154. 11.
